# Cytological Grading of Breast Tumors—The Human and Canine Perspective

**DOI:** 10.3389/fvets.2019.00283

**Published:** 2019-08-27

**Authors:** Krithiga Kuppusamy, Aarathi Rajan, Aarathy Warrier, Revathy Nadhan, Dipyaman Patra, Priya Srinivas

**Affiliations:** Cancer Research Program-6, Rajiv Gandhi Centre for Biotechnology, Thiruvananthapuram, India

**Keywords:** breast, canine, cytology, grading, human, tumor

## Abstract

Human breast cancers (HBCs) are one of the leading causes of global cancer death among women. Domesticated canines are the most affected domestic species with a prevalence rate of breast cancer more than three times in women. While the human cancer patients receive substantial diagnostic and treatment facilities, inadequacy in canine cancer care, calls for greater attention. Fine Needle Aspiration Cytology (FNAC) is comparatively simple, quick, and easily reproducible technique, which aids in pre-surgical diagnosis. In humans, FNAC has a standard protocol, the Robinson's grading system, which has high correlation with the established histological grading system of Scarff Bloom- Richardson. However, Canine Mammary Tumors (CMTs), which are known to be similar to HBCs in biological behavior and gene expressions, still bank on the histopathological methods for diagnostic purposes. This review sheds light on various factors that could be considered for developing a standard FNAC technique for CMT grading and analyzes its future perspectives.

## Introduction

In humans, breast cancer is the most common form of cancer that entrains the highest mortality rate in women (1). Diagnosis and prognosis of breast cancers rely on the triple wedge, mammography, clinical and laboratory examination of tumors. In general, histological type, tumor size, lymph node status, nuclear grade, proliferative index, and hormonal status are the key factors, which play a pivotal role in dictating prognosis. The National Institute of Health Consensus Conference on Adjuvant Therapy for Breast Cancers held in Bethesda, Maryland has concluded that all the above parameters are mandatory for the histopathological reports of Human breast cancers (HBCs) (2). In addition to these prominent factors, cytological grading plays a pivotal role in cancer diagnosis and it has also proved to be an important prognostic factor in predicting the metastasis-free and overall survival of the patients (3). Though histological analysis continues to be the gold standard for tumor grading, a pre-surgical cytological grading could be a better strategy as it is simple and easily reproducible. The Conference on the uniform approach to breast fine-needle aspiration biopsy at the National Cancer Institute, Bethesda has also suggested the inclusion of cytological grading of tumors in the histological reports (4).

As the constantly aggravating risk and augmenting deaths caused by HBCs continues to be a grave concern, the past few decades of cancer research has propelled toward the search for a model organism that can mimic and provide a better understanding of the underlying molecular pathology behind HBCs. The strikingly similar nature of Canine Mammary Tumors (CMTs) and HBCs in their biological behavior and molecular characteristics (5) along with the recurrence post-surgery and metastasis to distant organs like lungs and liver, make dogs an ideal model for studies involving HBCs. In canines, mammary tumors account for nearly 50% of the neoplastic cases in female dogs. The mammary tumors are mostly common in aged, intact female dogs, with a probable incidence as much as thrice that of women (6). While the promulgated rate of CMT being 42% across dogs of all breeds (7), about 50–70% of these tumors progress to malignancy (8, 9). This is most likely as a result of delayed diagnosis and poor prognosis of CMTs. The prognosis and treatment of the CMTs rely mainly on tumor staging and grading. Currently, the grading of CMTs are mostly carried out by histopathological analysis of tumor tissues, as there is no established system for Fine Needle Aspiration Cytology (FNAC) based grading.

The FNAC has been widely practiced in canines which uses Fine needle aspirates (FNA) collected via a less-invasive method to differentiate the benign tumors from the malignant ones. However, this doesn't involve any cytological grading. Even though there are numerous reports on the diagnosis of CMTs using cytology as a tool (10–12), their grading utilizing the cytological techniques has not been widely attempted. This review discusses and evaluates the possibility of recommending cytological grading system in canines, taking cues from the histopathological grading of canines and the well-established cytological grading system of human tumors.

## Cytological Grading for Human Mammary Carcinomas

In human breast cancer patients, FNAC is the prominent technique used for cytological grading of the breast cancers for pre-surgical diagnosis. FNAC was first used by Hayes Martin and Edward Ellis in 1930. This technique was lying dormant for nearly two decades until researchers from Scandinavian Karolinska University started exploring its utility for diagnosis of palpable breast masses. Until the 1990s, FNAC was predominantly used for the pre-surgical diagnosis of tumors, after which the Core Needle Biopsy (CNB) came into practice (13). In recent years, FNAC has been overshadowed in the developed countries by CNB (1).

Though CNB has the advantage of confirming calcifications seen during ultrasonography, thereby avoiding unnecessary surgical interventions for benign lesions (14), it is cumbersome as its sampling needs local anesthesia and expertise, since advertent sampling might result in either a non-representative sample, the hematomas or infections. However, in many countries, FNAC is still preferred over CNB, as the former is a simple, less invasive, and cost-effective method for breast cancer diagnosis (13).

Apart from differentiating the benign and malignant tumors, FNAC can be used as a powerful tool for cytological tumor grading too. Also, cytological grading has been reported to hold a statistical concordance of 66–76% with the histological grading (15). Thus, cytological grading in HBCs tends to be highly reproducible and at many instances, act as a substitute for histological grading to facilitate diagnosis and prognosis of HBCs. Neoadjuvant therapy based on cytological grading has gained impetus, as it has aided in the appropriate selection of suitable therapeutic strategies (16, 17).

The details about various three-tier cytological grading methods for human breast invasive ductal carcinoma are depicted in Table 1.

**Table 1:** Cytological grading methods for human breast cancers.

**Year, grading method**	**Parameters analyzed**	**Advantages**	**Disadvantages**
1980, Fisher's modified Black (18)	Nuclear characteristics like size, membrane contour, anisonucleosis, chromatin, and nucleoli	Powerful indicator of tumor aggressiveness when combined with histological type and race of patient (18)	Variation in nuclear size due to unavoidable air-drying during smear preparation, time consuming and more subjective (1)
1986, Mouriquand (19)	Cellular characteristics, nuclear features, nucleoli, and number of mitoses	Provide insights into disease free interval and early relapse, concordant with SBR (Scarff-Bloom—Richardson's) histological grading (19, 20)	Difficult to score and has low specificity (1, 21, 22). Disconcordance due to mitosis resulting in over-grading (23)
1990, Hunt (15)	Nuclear diameter, nuclear pleomorphism and presence of nucleoli. Similar to Fisher but there is scoring for nuclear features	The classification of tumors into high and low cytological grades which showed a close correlation with histological grade (24)	Insufficient categorization when compared to Modified Bloom Richardson histological grading (24). Nuclear features alone is not sufficient for grading (25). The other cellular details have also to be considered
1994, Robinson (26)	Cell dissociation, cell size, cell uniformity, nuclear margin, nuclear chromatin, and nucleolus	Simple, easier, and reproducible technique which correlated with the histological grade (21, 25)	Mild nuclear pleomorphism and cell dyscohesion could be reasons for discrepancies during grading, mitotic count not considered (17)
1994, Howell (3)	Tubule formation, nuclear pleomorphism, and mitoses	Predictable, reproducible with greater correlation with histology (1, 27)	Difficulty in identification of tubules and mitoses in cytological smears (1, 27)
1998, Yu (28)	Dyscohesion of cells, nuclear grading method used by Dabbs and Silverman (28, 29)	Statistical significance between dyscohesion and distant metastasis, slide fixation did not pose a problem	Nuclear grading and cellular dyscohesion score not combined, degree of dyscohesion not correlated with regional metastasis, no significant relationship between dyscohesion. and nuclear grading, mechanical interference due to smearing and difference in dyscohesion in different areas of the slide (30)
2000. Taniguichi (16)	Cell size, nuclear/cytoplasmic ratio, nuclear pleomorphism, nucleoli, chromatin granularity, density of chromatin, hyperchromatic, necrosis	Positive correlation with histological grade (16, 25); hyperchromatism; predictive of nodal metastasis ascertained using markers such as Estrogen receptors (ER) and MIB-1(16)	Has not been worked upon by any other study group (1) complexity of the method involving features like density and granularity of chromatin and nuclear/cytoplasmic ratio could be subjective
2003, Khan (31)	Pleomorphism, nuclear size and margin, nucleoli, naked tumor nuclei, mitotic count, cellularity, cell dispersion, lymphocytic response	Nuclear size statistically significant and pleomorphism plays a discriminatory role (25, 31)	Cell dispersion that varies due to smear preparation and mitotic count that is sparse in cytological smears due to the meager amount of aspirate used and fragility of the cells (1)
2006, Fan (29)	Nuclear grade, cellular dyscohesion, and bare atypical nuclei	Cytoprognostic score correlated with lymph node metastasis and ER and PR expression, correlated with histological grade (29)	Time consuming (1)

Each one of the cytological grading systems in HBCs mentioned in Table 1 has adopted a three-tier scoring; hence, all the parameters are scored at three levels depending on the cytomorphological characteristics. Thus, the final score obtained from the analysis aids in the grading of the tumors represented as Grade 1, 2, and 3.

An example of the widely used Robinson's cytological grading system is depicted in Table 2.

**Table 2:** Robinson's cytological grading of HBCs (26).

**Cytomorphology**	**Score 1**	**Score 2**	**Score 3**
Dissociation	Cells mostly in clusters	Mixture of single and cell clusters	Cells mostly single
Cell size	1–2 X Red blood cells (RBC) size	3–4 X RBC size	>5 X RBC size
Cell uniformity	Monomorphic	Mildly pleomorphic	Pleomorphic
Nucleoli	Indistinct	Noticeable	Prominent or pleomorphic
Nuclear margin	Smooth	Folds	Buds/clefts
Chromatin	Vesicular	Granular	Clumped and cleared

*Grade 1 = score 6–11; Grade 2 = score 12–14; Grade 3 = score 15–18*.

However, there are certain controversies over the application of cytological grading for the predictive analysis. Two schools of thought exist about the prediction of lymph node metastasis utilizing cytological grading, wherein some pathologists claim that there is a possibility of assessing the lymph node metastasis from the cytological grading of the HBCs (32, 33), while the others rule out any such correlation (14, 34).

## Mammary Tumors and Cytology in Canines

Mammary tumors in intact female dogs is a very common disease. The CMT reports state that complex carcinomas are the most commonly represented tumor type, followed by simple, solid, mixed, anaplastic carcinomas, and fibrosarcomas (35). The CMTs recorded most frequently are the malignant infiltrating ductal type tumors (36).

**Figure 1:**
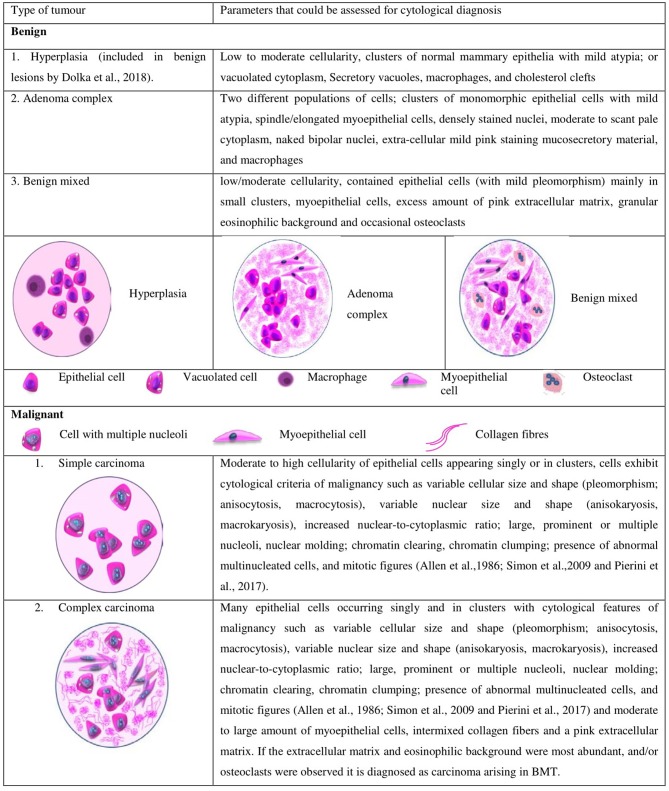
Different types of tumors and the cytological parameters currently used in canines for differentiating malignant and benign tumors.

FNAC can be used for the pre-surgical evaluation of the CMTs similar to that of HBCs (37, 38). However, it is utilized only for differentiating the benign tumors from the malignant ones, and not for cytological grading. The numerous cytological parameters evaluated for differentiation includes cellularity, variability in the size and shape of nucleus and cytoplasm, nuclear to cytoplasmic ratio, size and number of nucleoli, chromatin clumping and clearing, background components such as mucosecretory material, extracellular matrix, necrotic debris, inflammatory cells, and erythrocytes as opined by National Cancer Institute Fine-Needle Aspiration of Breast Workshop Subcommittees (4). The illustration of the cytological features used to differentiate the tumor types in canines is given in Figure 1.

### Cytological Grading of Canine Mammary Tumors: A Technique Less Explored

Cytological grading using FNAC has not been in the limelight as far as CMTs are concerned. Though the use of FNAC for cytological differentiation of CMTs and for analysis of their different cellular origins has been well-established (37, 39–41), there lies a lacuna on its application with regard to cytological grading. The cytological evaluation for the diagnosis of CMTs accounts for a sensitivity of 65–88% and specificity of 94–96% (37, 39) in comparison to the gold standard method of histopathological grading. The cytological grading system which is being utilized for the HBCs can also be applied to the CMTs for their diagnosis and prognosis; however, in spite of having an accuracy of about 88.5% (38), this technique has been less explored for the grading of CMTs.

### The Current Cytological Grading System in Canines

The literature review reveals that there are not any reports regarding the cytological grading system in canines using FNAC, except for a single publication (42). The numerous features analyzed in that study and the resultant score card adapted by them is depicted in Table 3.

**Table 3:** Scoring system for the cytological samples of CMT (42).

**Cytologic features**	**Score**			
	**0**	**1**	**2**	**3**
Cellularity		Scanty 10–20 cells/High Power Field (HPF- 40x objective)	Moderate 20–50 cells/HPF	Abundant > 50 cells/HPF
Cell dissociation (clusters)		Clusters > 5/10 HPF, single epithelial cells <25% of neoplastic cells	Clusters 3–4/10 HPF, single cells 25–75% of neoplastic cells	Clusters 1–2/10 HPF, mostly single cells more than 75%
Mucosecretory material, foamy macrophages	Absent/1–2 cells/HPF	Mild, 3–4 cells/HPF	Moderate, 5–10 cells/HPF	Abundant > 10 cells/HPF
Extracellular matrix	Absent	Mild	Moderate	Abundant
Necrotic debris	Absent	Mild	Moderate	Abundant
Inflammation	Absent, 1–2 single cells/HPF	Occasional, 3–4 cells/HPF	Moderate, 5–10 cells/HPF	Abundant > 10 cells/HPF
Red blood corpuscles/red blood cells	Absent/single 1–2 cells/HPF	Occasional 3–4 cells/HPF	Moderate 5–10 cells/HPF	Abundant > 10 cells/HPF

Their scheme was a modified version of the most acclaimed Robinson's grading system in HBCs. They reported that despite the similarities of CMTs with HBCs, mammary mixed tumors are more common in dogs than humans. This observation necessitates for an appropriate sampling technique and an experienced cytopathologist in aiding the diagnosis of CMTs. Previously, Dolka's group had already observed that cellularity and cytological background did not influence the overall survival period; however, Grade 2 and Grade 3 CMTs had a lower survival period and later begat to cancer associated mortality. Nevertheless, their study failed to prove that the cellular dissociation in cytological specimen could be a predictive factor for nodal metastasis in CMTs, in contrast to the report in HBCs (30). As there is an acute shortage of information regarding cytological grading in CMTs, establishment of a well-elucidated standard protocol requires cumulative effort from researchers all over to characterize and evaluate the grading system for canines.

## Discussion

Cytology has gained the status of a tool for pre-operative diagnosis of CMT with satisfactory sensitivity and specificity to differentiate benign and malignant lesions (37, 38). However, there are few impediments to resolve. The practical implications like the heterogenous nature of CMT, extensive necrosis, inflammation, and the challenge posed by mixed and complex tumors (10, 40) are noteworthy. These aspects have always made an ambiguous mark on the cytological diagnosis of CMT with the possibility of false-positives and false-negatives. There are always one or two snags like the cytological diagnosis of *in-situ* carcinoma, which gives a rather menacing image than real. Hence, cytological diagnosis and grading had setbacks. These issues however, can be addressed to a possible extent with consistent and regular practice by which cytology can get a better status in the realm of diagnosis. A keen methodical strategy that has to be carried out to improvise the cytological grading into an efficient diagnostic tool for CMTs. Several key factors have been enlisted in Table 4 that could be considered while standardizing an efficient protocol for the cytological grading of CMTs. The obstacles arising during the standardization procedures can be tackled by practical expertise and cross-references with the literature on HBCs.

**Table 4:** Factors that are to be considered while standardizing the protocol.

**S.no**.	**Factors to be considered**
1.	Intra and inter observer variation should be considered while standardizing the score card (43)
2.	Subjective variation due to the experience of cytopathologist in aspects of judging factors like pleomorphism, nuclear to cytoplasmic ratio, etc.
3.	Factors such as nuclear size, the appearance of chromatin, and cell clustering which are variable due to smear preparation and fixation (26)
4.	Sampling from different areas of a tumor should be done to avoid discrepancies
5.	It is necessary to compare the cytological grading with that of histopathological grading
6.	Regional lymph nodes can be examined adjunct to the tumor using FNAC to ascertain metastasis
7.	The case details and follow-up after surgery should be meticulously recorded
8.	The different grading systems used in the HBCs grading can be attempted as such or modified as per requirement
9.	FNAC does not give details about *in-situ* or invasive nature of carcinoma (14)
10.	The yield of cells in sclerotic masses will be less hampering the diagnosis
11.	The thread line of difference between the low-grade tumors and the benign counterpart should be critically analyzed

Though, the FNAC based grading systems has its advantages that would play an exquisite role in the diagnosis of CMTs, histopathological grading will continue to hold its eminence in the final diagnosis of the CMTs due to the paucity of work done in this realm (42). Several cytology-based systems which are used in the grading of invasive carcinomas of HBCs can be explored in CMTs in its naïve or modified forms, which would help to elucidate the most appropriate system of cytological grading for CMTs. The cytological grading system for canines as recommended in this review, given in Table 5, is a cumulative convergence of numerous factors discussed in different reports (3, 16, 26, 44).

**Table 5:** Suggested score card for grading canine mammary tumors with inputs from Robinson's, Khan's, Taniguchi's, Howell's grading systems and Bonzanini et al. (44).

**Cytological features**	**Score 1**	**Score 2**	**Score 3**
Cellularity/40x	10–20 cells	20–50 cells	>50 cells
Cell dissociation	Mostly in clusters	Cluster and single cells	Single cells
Syncytia formation/ 10X	1–2	2–4	More than 5
Cell size	1–2x red cell size	3–4x red cell size	≥5x red cell size
Cell uniformity	Monomorphic/mild pleomorphism	Moderate pleomorphism	Marked pleomorphism
Nuclear margin	Smooth	Irregular	Budding/clefts
Nuclear size	Uniform/ <3x Red cell size	3–5x red cell size	≥5x red cell size
Nucleoli	Indistinct	Noticeable	Prominent
Nuclear pleomorphism	Absent	Mild to moderate	Marked
Chromatin	Fine	Moderately granular	Coarse
Mitotic count/40X	Absent	1–2	More than 3
Naked tumor nuclei	<3x red cell size	3–5x red cell size	≥5x red cell size
Necrosis	Mild	Moderate	Marked
Inflammatory cells/40X	3–4 cells	5–10 cells	>10 cells
Tubule formation	Marked	Moderate	Mild/absent

*Grade 1- score 10–15; Grade 2- score 16–30; Grade 3- score 31–45. Syncytia- cell cluster with inconspicuous intercellular membrane and overlapping of nucleus. Tubule formation- cell cluster in which the cells are polarized forming tubule or gland like structures*.

**Figure 2:**
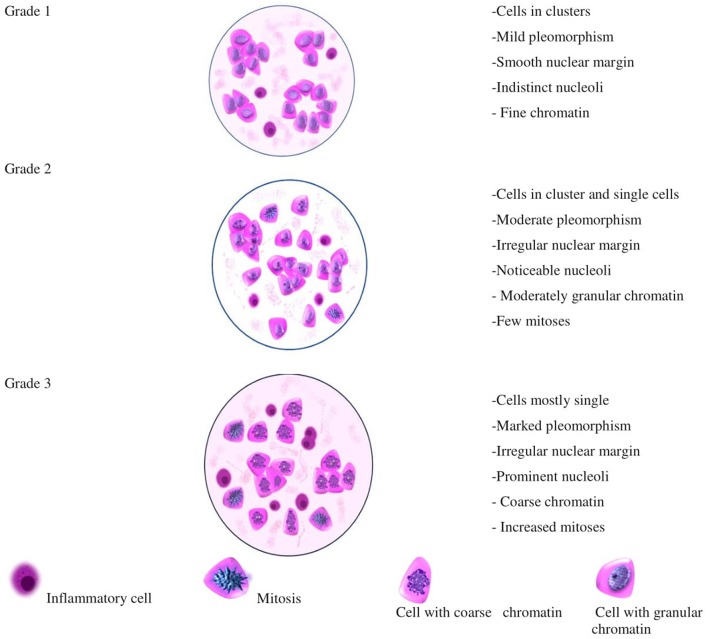
Illustration depicting the appearance of cells that could be considered for different cytological grades in CMT.

An illustration depicting the cells characterizing the numerous cytological grades has been represented in Figure 2, which will aid not only in the pre-surgical diagnosis, but also in the planning of neoadjuvant therapy. Further studies, involving molecular markers along with cytological grading, will help in authenticating the similarities between HBCs and CMTs, thus, making canines the unambiguous models for analyzing HBCs; as it has already been reported and established that canines can act as models for better understanding of HBCs and aiding the cancer research. Canines also develop cancers with an intact immune system similar to that of humans. Factors affecting the disease outcome, including tumor size, stage and lymph node invasion, are synonymous in HBCs and CMTs (5). A noteworthy feature in dogs and humans is the magnitude of genetic parity between them. For example, the breast cancer type I susceptibility gene (BRCA1), a prominent tumor suppressor gene whose mutation predisposes women to hereditary breast-ovarian cancers, has been reported to bear 84% sequence homology in dogs and humans. The higher magnitude of genetic similarity makes the dogs a better comparative genomics model over other species that are currently aiding human disease studies (45). Thereby, current times have seen a surge in canine cancer research as more and more number of clinical trial facilities are upcoming that would involve canine models. The University of Pennsylvania, Philadelphia, is housing a canine cancer clinical trial facility and recently, The Tallwood Canine Cancer Research Initiative at Jackson Laboratory (JAX) has made an initiative to bank and sequence canine tumors. The cytological grading in case of human subjects is well-established and has helped in the pre-surgical evaluation of the patients. The technique is less-invasive and reproducible and aids the diagnosis of the state of differentiation from benign to malignant one, thus serving as a pre-surgical pathological aid (1). An adequate grading system and resultant therapeutic regimes would help the substantial reduction in the financial loss and emotional stress of the pet owners.

This review provides insights into unleashing the possibilities of developing an efficient cytological grading system for CMTs, which would assist veterinary cytopathologists in diagnostic and prognostic purposes and serve our companion animal with the same diagnostic facility as that of the humans.

## Author Contributions

KK identified the lacunae and prepared the draft. AR helped in creating the illustrations. AW, RN, and DP helped in editing the review. PS conceived the concept of the review and approved the manuscript.

### Conflict of Interest Statement

The authors declare that the research was conducted in the absence of any commercial or financial relationships that could be construed as a potential conflict of interest.
